# Impedance Pneumography for Diagnosing and Monitoring Asthma in Young Children: A Systematic Review

**DOI:** 10.3390/children13020193

**Published:** 2026-01-29

**Authors:** Sama-Rafie Hammod, Fanny Kullberg, Marie Hauerslev, Kirsten Skamstrup Hansen, Bo Chawes

**Affiliations:** 1 Department of Pediatrics, Herlev and Gentofte Hospital, University of Copenhagen, 2730 Herlev, Denmark; phz355@alumni.ku.dk (S.-R.H.); marie.hauerslev@regionh.dk (M.H.); kirsten.skamstrup.hansen@regionh.dk (K.S.H.); fanny.kullberg@gmail.com (F.K.); 2 Allergy Clinic, Herlev and Gentofte Hospital, University of Copenhagen, 2820 Gentofte, Denmark; 3 Department of Clinical Medicine, Faculty of Health and Medical Sciences, University of Copenhagen, 2200 Copenhagen, Denmark

**Keywords:** asthma diagnostics, children, asthma, wheeze, impedance pneumography

## Abstract

**Background:** Impedance pneumography (IP) is a non-invasive technique for assessing tidal breathing in young children and enables home-based recordings without active patient cooperation. By deriving tidal breathing flow–volume (TBFV) curves and indices such as the expiratory variability index (EVI), IP has been proposed as a tool for identifying obstructive breathing patterns and monitoring airway function in early childhood. However, its clinical role in asthma and wheezing disorders has not been systematically evaluated. This review aimed to assess the evidence of IP in differentiating healthy children from those with asthma or recurrent wheeze, in reflecting treatment-related changes or acute bronchial obstruction, and in relation to other lung function tests. **Methods:** A systematic literature search of PubMed, Medline, Embase, and the Cochrane Library databases was conducted on 5 January 2026. Original studies using IP in children aged 0–7 years with asthma or wheeze were eligible. Study selection followed PRISMA guidelines, and risk of bias (RoB) was assessed using the Newcastle–Ottawa Scale (NOS). Due to substantial heterogeneity in study design, populations, and outcome measures, results were synthesized narratively. **Results:** Five studies were included, with a total of 376 participants aged 0.5–7.0 years. Three studies reported significantly lower EVI values and TBFV profile variation in children with asthma or recurrent wheeze compared with healthy controls. Two studies found an association between EVI and markers of airway obstruction. Changes in IP measures following inhaled corticosteroid treatment or medication withdrawal were reported, suggesting sensitivity to treatment-related changes. However, study quality was moderate to low, with small sample sizes, heterogeneous outcome definitions, and limited diagnostic validation. **Conclusions:** Current evidence suggests that IP-derived indices, particularly EVI, capture clinically relevant features of obstructive breathing patterns in young children and may be useful for longitudinal monitoring of airway function. However, evidence supporting a diagnostic role for IP in childhood asthma remains limited. Larger, independent, and methodologically robust studies are needed before IP can be integrated into routine clinical practice.

## 1. Introduction

Asthma is the most common chronic respiratory disease in children, particularly in early childhood. It is characterized by wheezing, coughing, and breathing difficulties and affects approximately one in ten children worldwide, although prevalence varies substantially across countries [1,2]. In preschool children, however, respiratory symptoms such as wheeze and cough are even more common, with more than 30% experiencing at least one episode [3]. These symptoms are not specific to asthma and may also reflect transient wheezing disorders or episodes of acute bronchial obstruction, most often related to viral respiratory tract infections. Consequently, particularly in preschool children, the distinction between asthma, recurrent wheeze, and isolated obstructive episodes is often unclear, as these entities represent overlapping but distinct clinical and pathophysiological conditions [4].

In clinical practice, asthma diagnosis in young children is often based on symptom history, clinical examination, and response to asthma medication, rather than on objective lung function testing. This carries a risk of both over- and underdiagnosis and may contribute to variability in reported asthma prevalence across regions [5,6]. Objective assessment of lung function in young children remains challenging, as conventional lung function tests such as spirometry, impulse oscillometry (IOS), and whole-body plethysmography require patient cooperation and are primarily validated for use in children aged three years and older [7,8,9]. Consequently, children below this age often lack access to reliable and objective lung function assessment in routine clinical practice [7,9,10].

These limitations have driven interest in alternative, non-invasive methods for assessing respiratory function in young children. Impedance pneumography (IP) has emerged as a potential tool for continuous, non-invasive respiratory monitoring in children aged 0–7 years, allowing monitoring in the home environment without active cooperation [11,12,13]. Using four electrodes placed on the thorax and armpits, IP measures changes in thoracic impedance to derive tidal breathing flow–volume (TBFV) curves during sleep [14]. Reduced variability in specific parts of the expiratory segment has been shown to reflect airway obstruction [15] and forms the basis of the expiratory variability index (EVI).

IP has been proposed as a tool for identifying obstructive breathing patterns, monitoring disease activity, and evaluating treatment response in young children. However, it remains uncertain whether IP can reliably distinguish healthy children from those with asthma or recurrent wheeze, or whether its primary value lies in longitudinal monitoring rather than diagnostic classification.

The aim of this systematic review was to evaluate the existing evidence on IP in young children with asthma-like symptoms, specifically assessing whether IP-derived measures can differentiate between healthy and symptomatic children, reflect airway obstruction and treatment response, and relate to other established lung function tests. Given the limited number of available studies, asthma, recurrent wheeze, and obstructive episodes are considered together within this review, while acknowledging their clinical heterogeneity.

## 2. Materials and Methods

### 2.1. Protocol and Registration

The study protocol was developed based on the Cochrane Handbook for Systematic Reviews of Interventions and reported in accordance with the Preferred Reporting Items for Systematic Reviews and Meta-Analyses (PRISMA) guidelines. The review protocol is registered in PROSPERO with the ID CRD42024621515.

### 2.2. Literature Search

#### Search Technique

Four databases (PubMed, Medline, Embase, and the Cochrane Library) were systematically searched on 5 January 2026, without any restrictions regarding publication date or country of origin. The search strategy was performed independently by two reviewers (SRH and FK) and included the following search terms: ((((child*) OR (toddler)) OR (infant)) OR (preschool)) AND (((impedance pneumography) OR (“expiratory variability index”)) OR (“tidal breathing flow volume”)).

### 2.3. Selection of Studies

Titles and abstracts identified through the literature search were independently screened by SRH and FK according to the predefined inclusion criteria. Studies considered potentially eligible were subsequently assessed in full text to determine final eligibility. Any disagreements were resolved through discussion and consensus. Study selection was managed using Covidence (Veritas Health Innovation Ltd., Melbourne, Australia), and all screening decisions were documented to allow completion of a PRISMA flow diagram.

### 2.4. Inclusion and Exclusion Criteria

Studies were included according to the following predefined inclusion criteria:

- Original studies.
- Studies concerning children aged 0–7 years of any sex or ethnicity with symptoms typical of asthma (e.g., coughing, wheezing, breathing difficulties).
- Lung function assessed by IP.
- Studies published in English.

Predefined exclusion criteria were as follows:

- Systematic reviews, abstracts, protocols, and unpublished studies.
- Studies lacking full text.
- Children with lung diseases other than asthma or wheeze.

### 2.5. Outcomes

#### 2.5.1. Primary Outcome

EVI in healthy children versus children with asthma or wheeze.

#### 2.5.2. Secondary Outcomes

- EVI in relation to asthma treatment response.
- EVI in relation to acute bronchial obstruction.
- EVI in relation to other lung function tests (whole–body plethysmography, IOS, and spirometry).
- Other TBFV indices in healthy children versus children with asthma or wheeze, and in relation to treatment response and acute bronchial obstruction.

### 2.6. Data Extraction

The following data were extracted from the included studies:

- First author
- Year of publication
- Country and study period
- Study design
- Aim
- Inclusion criteria
- Medication use
- Number of participants
- Sex (male)
- Age group (median and range)
- IP parameters (EVI, TBFV)
- Other lung function tests
- Results

Data were independently extracted by two reviewers (SRH and FK) from all included studies. Any discrepancies were resolved through discussion. When necessary, study investigators were contacted to clarify or confirm unclear information. No automation tools were used in the data extraction process.

### 2.7. Risk of Bias Assessment

The risk of bias (RoB) in the included studies was assessed using the Newcastle–Ottawa Scale (NOS) for non-randomized studies. The NOS assesses three domains: selection of study groups, comparability of groups, and outcome (for cohort studies) or exposure (for case–control studies). Each study can receive a maximum of nine stars, with higher scores indicating lower risk of bias. RoB assessment was performed independently by SRH and FK. Any discrepancies in scoring were resolved through discussion with a third author (BC).

### 2.8. Data Synthesis

All included studies were examined for data relevant to each predefined outcome. To determine eligibility for each synthesis, reported intervention, population, and outcome characteristics were compared with the prespecified synthesis groups. Relevant data were extracted, and study authors were contacted when data were unclear or missing.

Study findings were summarized narratively and presented in structured tables. Although a meta-analysis was initially planned, substantial heterogeneity in study design, populations, and outcome reporting precluded quantitative synthesis; therefore, a narrative synthesis was conducted.

As statistical pooling was not feasible, the potential risk of bias due to missing or selectively reported results was assessed qualitatively using the selective reporting domains of the NOS. The certainty of the evidence was not formally assessed, as heterogeneity in study designs and outcome measures prevented the calculation of comparable effect estimates.

## 3. Results

### 3.1. Study Selection

The initial search identified 318 studies. After the removal of 172 duplicates, 146 unique articles remained. These were screened by title and abstract, resulting in the exclusion of 122 articles that did not use IP or did not focus on asthma or wheeze. Full-text assessment was performed on the remaining 24 articles, of which 19 were excluded because they did not meet the age criteria, did not focus on IP, were conference abstracts, or had outcomes, settings or comparators that did not align with the predefined inclusion and/or exclusion criteria.

**Figure 1:**
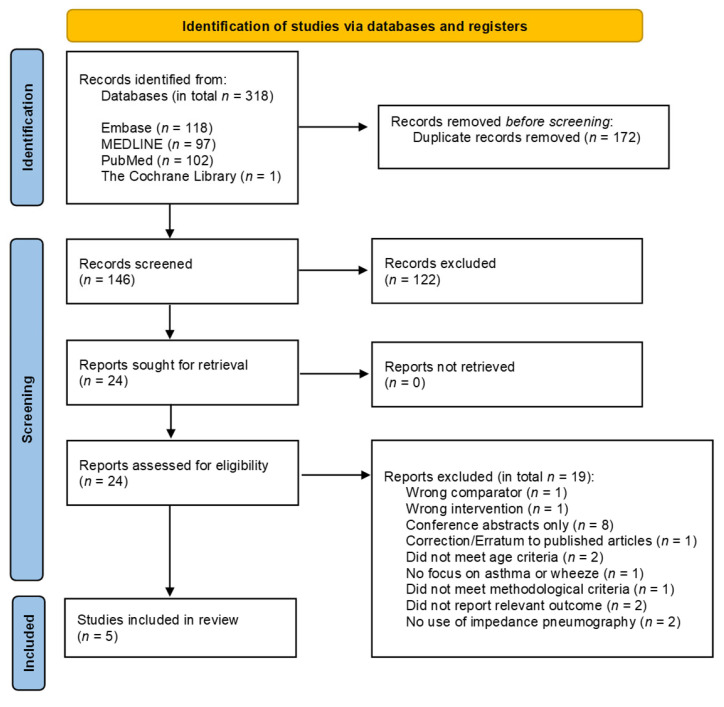
PRISMA flow chart for study selection.

In total, five articles were included in the review. The PRISMA flow diagram is presented in Figure 1. One additional study was identified through contact with the authors of another study. However, as this study had not yet been published, it was not included in this review. Further details on studies excluded at the full-text assessment are provided in the Supplementary Materials.

### 3.2. Study Characteristics

Of the five included studies, three were conducted at university hospitals in Finland [16,17,18], one in Croatia [11], and one in both Croatia and Finland [15]. The total number of participants was 376 children aged 0.5–7.0 years, with usable data from 345 participants. In three studies, sex distribution was reported, with the proportion of males ranging from 39% to 77% [11,16,17]. Further study characteristics are presented in Table 1 and Table 2 and results are presented in Table 3 and Table 4.

A meta-analysis was initially planned but could not be performed due to substantial heterogeneity across studies with respect to study design, populations, measurement methods, and outcomes. Therefore, the findings are presented using a narrative synthesis approach.

### 3.3. EVI for Differentiation Between Healthy and Asthmatic Children

Of the five included studies, three compared IP assessments among healthy children and children with asthma or recurrent wheeze [11,15,17]. Two of these studies reported outcomes using EVI, while one assessed variability in TBFV profiles. Both studies reporting EVI found significantly lower values in children with asthma or recurrent wheeze than in healthy controls [11,17]. However, only one study reported numerical EVI values [11] whereas the other presented results graphically using boxplots, precluding extraction of absolute values.

In the first study, Seppä et al. [11] reported significantly lower EVI values in children hospitalized due to acute bronchial obstruction (*n* = 30, 1.3–5.3 years) than in healthy controls (*p* < 0.0001). Hospitalized children had a median EVI of 12.4 (IQR 7.7) two days before discharge from hospital and an EVI of 14.9 (IQR 5.6) one day before discharge, while healthy controls had an EVI of 17.4 (IQR 3.8). No significant differences in EVI were observed between healthy controls and patients two and four weeks after hospital discharge.

In the second study [17], children with recurrent wheeze (*n* = 70, 1.0–5.6 years) received inhaled corticosteroids (ICS) for three months. IP was performed eight days before ICS withdrawal and again 10 and 25 days after withdrawal. EVI values were significantly lower in patients at all three time points than in healthy controls (*p* < 0.01). Furthermore, an EVI below the 10th percentile of controls (EVI = 14.0) 25 days after ICS withdrawal had a sensitivity of 46% and specificity of 90% for distinguishing children with recurrent wheeze from healthy controls [17]. The article did not present EVI values numerically for children with recurrent wheeze, but graphically as boxplots only.

In the third study [15], IP was recorded four weeks after withdrawal of a three-month course of ICS in children with a history of three physician-witnessed lower airway obstruction episodes (*n* = 61, 0.9–5.7 years). Variability in the TBFV curve was reduced in the 15–45% segment of exhaled volume in patients compared with healthy controls (*n* = 39, 1.5–6.0 years) (*p* < 0.001). No significant differences were observed in the TBFV curves in the first half of inspiration or the very early part of the expiration between patients and controls [15].

### 3.4. EVI and Asthma Treatment Response

Two of the five included studies evaluated EVI in relation to ICS treatment [16,17]. One study reported numerical EVI values [16], whereas the other presented EVI graphically using boxplots.

In the study by Seppä et al. [17], EVI was measured at home over three consecutive nights in healthy controls and in children with recurrent wheeze at three time points: eight days before ICS withdrawal (baseline), and 10 days (2 W) and 24 days (4 W) after withdrawal. A significant within-patient reduction in EVI was observed at 4 W compared with both 2 W (*p* = 0.007) and baseline (*p* = 0.003), whereas no significant difference was found between baseline and 2 W.

In the study by Burman et al. [16], EVI was assessed over two consecutive nights before and two time points after initiation of ICS treatment, namely baseline, day 43, and day 68, in children with physician-diagnosed asthma. This study demonstrated a significant improvement in EVI from baseline during ICS treatment (*p* = 0.028), with an EVI of 17.5 (15.2–18.9) at baseline, 18.4 (17.2–30.0) at day 43 after ICS initiation, and 17.9 (17.1–19.6) at day 68.

### 3.5. EVI and Acute Bronchial Obstruction

Two of the five included studies investigated acute bronchial obstruction [11,17]. In one of the studies [17], EVI was used to monitor children with recurrent wheezing one week (baseline) before discontinuation of a three-month ICS treatment trial, and again 10 days (2 W) and 24 days (4 W) after withdrawal. At the 4 W follow-up, children who had used a bronchodilator (BD) during the same day or evening (BD+, *n* = 17), with bronchodilator use serving as a proxy for acute bronchial obstruction, had significantly lower EVI values compared with children who had not used a BD (BD–, *n* = 44) (*p* = 0.02). The area under the curve (AUC) for this distinction was 0.88 (95% CI: 0.75–0.99) in the BD+ group and 0.74 (95% CI: 0.65–0.82) in the BD– group. This difference was not significant at baseline (*p* = 0.38) and was only borderline significant at the 2 W follow-up (*p* = 0.07). EVI results were reported graphically, without numerical values.

Furthermore, an abnormal reduction in EVI between baseline and 4 W—defined as a decrease exceeding the normal range of night-to-night variability observed in controls in the study—was more common in children who required BD. Such a reduction was observed in 63% of BD+ compared with only 15% of BD– (*p* = 0.001) [17].

In the other study addressing this endpoint [11], EVI was used to monitor children hospitalized for acute bronchial obstruction over several nights during hospitalization and again at two and four weeks after discharge. EVI values were significantly lower during the inpatient period compared with follow-up assessment at two and four weeks (pooled inpatient results: *p* = 0.0002 and *p* < 0.0001, respectively). A median EVI of 14.9 (IQR 5.6) was reported one day before discharge, which improved to 16.4 (IQR 4.4) at the two-week follow-up after discharge, and further to 17.0 (IQR 5.5) at the four-week follow-up.

EVI also differed significantly across three categories of chest auscultation findings during hospitalization and at the follow-up visits (*p* = 0.0001) [11]. Children with normal auscultation findings had a mean EVI of 17.5 (4.9), whereas those with wheezing and/or crackles had a significantly lower mean EVI of 11.4 (6.8), corresponding to an AUC of 0.81 (95% CI: 0.71–0.91). In children with prolonged expiration only, the mean EVI was 15.6 (7.4), with an AUC of 0.67 (95% CI: 0.55–0.79). Eighty-three percent of abnormal auscultation findings were reported during hospitalization. Finally, EVI showed a significant negative correlation with the number of days until discharge, with lower EVI values associated with a longer hospital stay (r = –0.38, *p* = 0.004).

### 3.6. EVI and Other Lung Function Tests

Three of the five included studies compared IP with other lung function tests. In the first study [18], lung function was assessed using maximal flow at functional residual capacity (V’maxFRC), measured by the rapid thoracic compression technique (BabyBody Masterscreen, Jaeger Ltd., Germany) under sedation in children with recurrent respiratory symptoms (*n* = 43, 6.0–23.0 months). Fractional exhaled nitric oxide (FeNO) and airway hyperresponsiveness (AHR) to methacholine were also assessed in a subgroup of 14 children. EVI showed a moderate positive correlation with V’maxFRC (Pearson correlation *r* = 0.52, *p* = 0.002), whereas no significant associations were observed between EVI and either AHR or FeNO.

In the second study [11], IOS was compared with IP in hospitalized children. No significant differences were observed in resistance at 5 Hz (R5) percent predicted between the inpatient period and subsequent follow-up visits (*p* = 0.83). Similarly, no significant difference was observed in bronchodilator reversibility of R5 between these time points (*p* = 0.17). Furthermore, EVI was not significantly associated with baseline R5 percent predicted (*r* = –0.13, *p* = 0.28) or with the degree of bronchodilator reversibility of R5 (*r* = –0.05, *p* = 0.75).

In the third study [16], IP was compared with IOS, bronchodilator reversibility, and a free-running test. Correlation analyses did not reveal any significant associations between EVI and IOS parameters, including R5 (*r* = –0.038, *p* = 0.835) or reactance at 5 Hz (X5, *r* = 0.217, *p* = 0.267).

### 3.7. Risk of Bias Assessment

**Figure 2:**
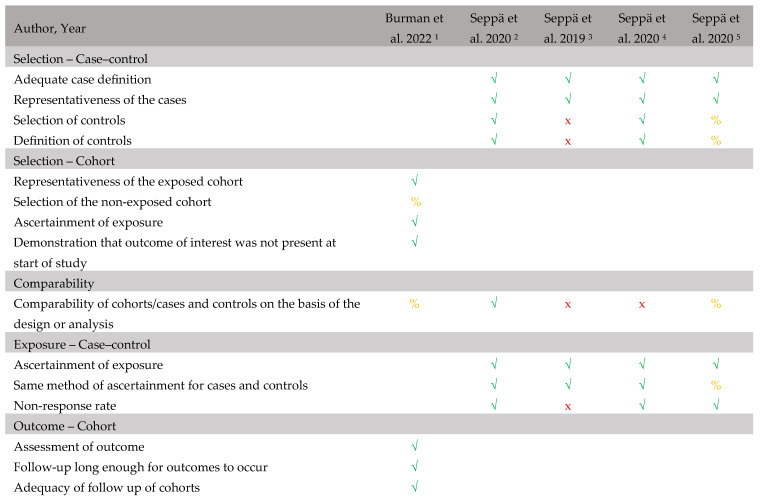
The Newcastle–Ottawa Scale for assessing risk of bias. Symbols: *√* = criterion fulfilled, x = criterion not fulfilled, % = not applicable. References: ^1^ [16], ^2^ [11], ^3^ [15], ^4^ [17], ^5^ [18].

The RoB assessment of the five included studies using the NOS is presented in Figure 2. Overall, the studies were judged to be of low to moderate methodological quality, with several recurring limitations identified.

Selection bias: Most studies clearly defined their case populations and demonstrated adequate representativeness. However, one study [15] did not clearly describe the selection or definition of the control group, indicating an increased risk of selection bias. In addition, this study included patient and control groups from different countries, with patients recruited in Finland and controls in Croatia. In the study by Burman et al. [16], a substantial risk of bias was introduced by the exclusion of a considerable number of children (*n* = 21) from the analysis due to missing data or loss to follow-up.

Comparability: Only one study [11] adequately controlled for potential confounding factors through study design or analysis, highlighting limited comparability across the studies.

Ascertainment of exposure and outcome: Most studies used consistent and appropriate methods for exposure assessment and applied similar procedures across cases and controls, reducing the risk of bias in this domain. In cohort studies, outcome assessment and follow-up procedures were generally well reported, particularly in the study by Burman et al. [16], which had clearly documented follow-up procedures and duration. However, in several studies, the number of nights with IP recordings differed between cases and controls [11,15,17], which may have introduced measurement bias.

Non-response bias: Four studies reported information relevant to non-response [11,16,17,18]. One study [16] identified differences in sex and age between participants with complete and incomplete IP, indicating potential non-response bias, although the impact on IP outcomes was not examined. The remaining studies reported technical reasons for missing data but did not assess whether non-responders differed systematically from responders.

Overall, the primary sources of bias were limited control of confounding variables, unclear selection and comparability of control groups in some studies, and incomplete reporting. These limitations should be considered when interpreting the findings, particularly regarding causality and generalizability. An additional potential source of bias is authorship overlap, as four out of five included studies share the same first author. Furthermore, three out of five studies were published as research letters, which provide limited methodological detail and restricted data extraction.

## 4. Discussion

This systematic review examined the current evidence for IP in the assessment of airway obstruction in young children aged 0–7 years with asthma or recurrent wheeze. Across the five included studies, IP-derived measures, particularly EVI, were consistently lower in children with asthma or recurrent wheeze compared with healthy controls. EVI was also associated with treatment response and reflected episodes of acute bronchial obstruction. Collectively, these findings suggest that IP captures clinically relevant features of obstructive breathing patterns in early childhood. However, given the limited number of included studies and substantial methodological heterogeneity, IP should currently be regarded as a promising but still investigational tool, with stronger support for monitoring than for definitive diagnosis.

Although a meta-analysis was initially planned, it was not feasible because of substantial differences between studies in design, populations, outcome definitions, and reporting. The results are therefore presented as a narrative synthesis.

### 4.1. Principal Findings for Primary and Secondary Endpoints

The primary aim of this review was to assess whether EVI can differentiate between healthy children and those with asthma or recurrent wheeze. Across the included studies, EVI values were consistently lower in children with asthma or wheeze than in healthy controls, suggesting that IP captures features of airway limitation in preschool children—a population in whom conventional lung function testing is often impractical [9]. However, the EVI threshold proposed in one study to discriminate healthy children from those with recurrent wheeze showed high specificity but relatively low sensitivity, limiting its usefulness as a screening tool [17]. Although the association of lower EVI in children with asthmatic symptoms was observed across heterogeneous study designs and populations, small sample sizes and non-standardized asthma definitions further limit the strength and generalizability of the evidence.

Regarding the secondary outcomes, IP was able to reflect acute airway obstruction and treatment-related changes in airway function [11,16,17], supporting its potential role as a monitoring tool in young children with asthma-like symptoms. However, in one study, airway obstruction was inferred indirectly through parental administration of bronchodilator medication at home, representing a surrogate outcome that weakens causal interpretation [17].

Although an EVI cut-off value below 14 was proposed as abnormal [11,16,17], one of the articles presented EVI data only graphically, without reporting absolute values [17], and in the remaining studies, EVI results were frequently above this threshold. This variability suggests that EVI may be more suitable for longitudinal monitoring of airway function than as a standalone diagnostic threshold.

Supportive evidence is provided by studies using alternative IP-derived indices. Gracia-Tabuenca et al. [12] demonstrated sleep- and time-dependent changes in several expiratory TBFV indices following ICS withdrawal in preschool children with recurrent wheeze, consistent with increased airflow limitation. Similarly, Seppä et al. [19] reported reduced respiratory chaoticity and altered overnight expiratory flow–volume curve characteristics in children at higher risk of persistent asthma compared with those at lower risk.

Overall, these studies suggest that IP is sensitive to clinically relevant changes in expiratory breathing patterns associated with disease activity, although they do not provide direct diagnostic validation.

### 4.2. Comparison with Other Lung Function Tests

Compared with conventional lung function methods such as spirometry or IOS, IP offers important practical advantages for young children, as it requires no active cooperation and enables repeated overnight home recordings [9,11,17,18]. However, unlike IOS or spirometry, IP does not provide direct measurements of lung volumes, airflow, airway resistance, or airway system compliance. Instead, IP indices such as EVI should be regarded as markers of breathing pattern variability, limiting their current application to pattern recognition and trend monitoring rather than direct physiological quantification [16].

Across the included studies, EVI did not correlate significantly with IOS parameters such as R5 or bronchodilator reversibility [11,16]. Furthermore, EVI was not associated with FeNO levels or AHR, although an association with V’maxFRC was reported in one study [18]. This lack of correlation suggests that EVI may capture aspects of airway dysfunction that differ from those assessed by IOS or inflammatory markers, rather than serving as a direct surrogate for these measures. No studies directly compared IP with spirometry or whole-body plethysmography.

Taken together, these findings indicate that IP may provide complementary information to existing lung function tests, particularly in very young children. However, the limited number of comparative studies underscores the need for further research to clarify how IP-derived indices relate to established physiological measures.

### 4.3. Clinical Implementation and Practical Limitations of Impedance Pneumography

From a practical perspective, IP offers several advantages that are particularly relevant in pediatric respiratory care. Measurements are non-invasive, require minimal cooperation, can be performed in the home environment, and allow continuous overnight assessment of breathing patterns. These features support the feasibility of repeated measurements in young children.

Nonetheless, several practical limitations must be considered when assessing IP measurements. Reliable recordings depend on stable electrode-to-skin contact and correct electrode placement over prolonged periods [14]. Across the included studies, failure rates of IP recordings ranged from a few percent to more than 20%. Failures were mainly due to electrode detachment, motion artefacts, or device malfunction.

In addition, IP indices are known to be influenced by factors unrelated to airway obstruction. Body position, sleep stage, and movement can alter tidal breathing patterns, potentially confounding interpretation [12,13]. Moreover, because IP does not provide direct measurements of airflow or resistance, its current clinical applicability appears best suited to longitudinal monitoring and identification of changes over time rather than diagnostic classification.

Therefore, before IP can be integrated into routine clinical practice, clear data quality criteria, standardized measurement protocols, and a better understanding of meaningful thresholds and confounding factors are required.

### 4.4. Methodological Considerations and Risk of Bias

All included studies had a moderate to high risk of bias, primarily due to small sample sizes, incomplete data reporting, and notable methodological differences. The use of EVI as the sole outcome in most studies, despite the availability of alternative metrics such as overnight changes in expiratory flow–volume minimum curve shape correlation and measures of respiratory chaoticity, or more detailed TBFV analyses, may have limited the interpretability and comparability of the findings [12]. Furthermore, four of the five studies shared the same first author, introducing a potential risk of publication or reporting bias. Three studies were published as research letters with limited methodological detail and incomplete participant descriptions, further constraining reproducibility and external validity [15,16,18].

RoB was assessed using the NOS, as all included studies were observational or cross-sectional in design. Although the NOS is a widely used and validated tool for assessing general methodological quality in observational studies, it is not specifically designed to evaluate diagnostic accuracy or prognostic performance. Consequently, aspects that are particularly relevant for diagnostic validation may not be fully captured by this tool. This limitation should be considered when interpreting the findings of this review, particularly regarding the potential diagnostic role of IP. Future studies and systematic reviews focusing on diagnostic validation may benefit from the use of more specialized RoB assessment tools tailored to diagnostic research.

### 4.5. Strengths and Limitations of the Review

Strengths of this review include a prospectively registered protocol, predefined primary and secondary endpoints, adherence to PRISMA guidelines, and a structured assessment of RoB. However, the small number of included studies, moderate to high risk of bias, lack of long-term follow-up, and substantial heterogeneity precluded a meta-analysis and limited conclusions regarding the diagnostic and prognostic value of IP. Attempts to obtain additional unpublished data were unsuccessful, and one potentially relevant study remains unpublished at the time of this review.

## 5. Conclusions

This systematic review indicates that IP, particularly through indices such as EVI, has potential as a non-invasive tool to capture obstructive breathing patterns in children aged 0–7 years. The available evidence suggests that IP-derived measures may be useful for monitoring airway function and detecting changes related to disease activity or treatment response in young children, rather than serving as a standalone diagnostic tool.

However, the current evidence base is limited by a small number of heterogeneous observational and cross-sectional studies with moderate to high risk of bias.

Further large, independent, prospective studies using standardized measurement protocols and well-defined populations are needed to clarify the role of IP in pediatric respiratory care. Comparative studies evaluating IP alongside conventional lung function tests will also be essential to determine its complementary value and to define its potential role in clinical monitoring and decision-making in early childhood.

## Figures and Tables

**Table 1:** Study design and population characteristics of included studies.

First Author Year	Country and Study Period, Case/Control	Study Type	Number of Participants (*n*) Case/Control	Dropouts/Missing Data (*n*) Case/Control	Male (%) Case/Control	Age in Years (Median, Range) Case/Control
Burman et al. 2022, [16]	Finland, NA	Observational study, research letter	53/NC	21/NC	66/NC	5.1 (IQR 4.6–5.8) */NC
Seppä et al. 2020, [11]	Croatia, 2018/2017	Prospective observational study	30/39	NA Failure in 19–20%/11% of recordings	77/39	2.8 (1.3–5.3)/4.1 (1.5–5.9)
Seppä et al. 2019, [15]	Finland, 2014– 2017/Croatia, 2017	Cross-sectional study, research letter	61/39	NA	NA	2.5 (0.9–5.7)/4.3 (1.5–6.0)
Seppä et al. 2020, [17]	Finland, 2014–2017/2018–2019	Prospective observational cohort study	70/41	2/1	67/55	2.4 (1.0–5.6)/3.7 (1.0–5.9)
Seppä et al. 2020, [18]	Finland, 2014–2017/NC	Observational study, research letter	43/NC	7/NC	NA	12.8 months (6.0–23.0 months)/NC

Abbreviations: IQR, interquartile range; NA, not available; NC, no control group. Age is reported in years unless otherwise specified. * The study reported age dispersion as an interquartile range rather than a total range.

**Table 2:** Impedance pneumography parameters, comparison tests, and inclusion criteria of included studies.

First Author Year	IP-Parameters	Lung Function Tests in Comparison	Inclusion Criteria Case/Control	Medication
Burman et al. 2022, [16]	EVI	IOS (R5, X5)	Physician-diagnosed asthma and clinical need to start ICS treatment/NC	6-month ICS
Seppä et al. 2020, [11]	EVI	IOS (R5, BDR)	Acute airway obstruction or asthma exacerbation requiring inpatient treatment/Healthy children with low risk of asthma	SCS + ICS + SABA at hospital admission, ICS at discharge
Seppä et al. 2019, [15]	Variability in TBFV profiles	NA	Three physician-witnessed lower airway obstructions/Healthy children with low risk of asthma	3-month ICS
Seppä et al. 2020, [17]	EVI	NA	Recurrent obstructive bronchitis/Healthy children with low risk of asthma	3-month ICS
Seppä et al. 2020, [18]	EVI	Rapid thoracic compression under sedation (V’maxFRC), methacholine test (AHR), FeNO	Children referred to infant lung function test due to troublesome respiratory symptoms/NC	NA

Abbreviations: AHR, airway hyperresponsiveness; BDR, bronchodilator response; EVI, expiratory variability index; FeNO, fractional exhaled nitric oxide; ICS, inhaled corticosteroids; IOS, impulse oscillometry; IP, impedance pneumography; NA, not available; NC, no control group; R5, resistance at 5 Hz; SABA, short-acting beta-agonist; SCS, systemic corticosteroids; TBFV, tidal breathing flow–volume; V′maxFRC, maximal flow at functional residual capacity; X5, reactance at 5 Hz.

**Table 3:** Summary of findings from studies using impedance pneumography in children with asthma or wheeze.

First Author Year	IP in Healthy Children vs. Children with Asthma/Wheeze	IP and Treatment Response	IP and Acute Bronchial Obstruction	IP vs. Other Lung Function Tests
Burman et al. 2022, [16]	NA	EVI improved from baseline after ICS initiation (*p* = 0.028).	NA	EVI not correlated with R5 or X5.
Seppä et al. 2020, [11]	EVI lower in children with acute airway obstruction vs. healthy during hospital admission (*p* < 0.0001).	NA	EVI lower during inpatient period than 2W and 4W follow-up (*p* = 0.0002 and *p* < 0.0001). EVI correlated with number of days to discharge (*r* = –0.38, *p* = 0.004).	EVI not correlated with R5 or BDR.
Seppä et al. 2019, [15]	Reduced TBFV variability in wheezing children vs. healthy at 15–45% exhaled volume (*p* < 0.001).	NA	NA	NA
Seppä et al. 2020, [17]	EVI lower in wheezing children than in healthy children across all three time points (*p* < 0.01).	EVI lower at 4W compared to 2W (*p* = 0.007) and baseline (*p* = 0.003) after ICS withdrawal.	EVI lower in children who used BD at 4W follow-up (*p* = 0.02). Abnormal reduction of EVI (>3.3) * from baseline to 4W more frequent in children that used BD vs. children that did not use BD (*p* = 0.001).	NA
Seppä et al. 2020, [18]	NA	NA	NA	EVI correlated with V’maxFRC (*r* = 0.52, *p* = 0.002). No correlation to AHR or FeNO.

Abbreviations: AHR, airway hyperresponsiveness; BD, bronchodilator; BDR, bronchodilator response; EVI, expiratory variability index; FeNO, fractional exhaled nitric oxide; ICS, inhaled corticosteroids; IP, impedance pneumography; NA, not available; R5, resistance at 5 Hz; TBFV, tidal breathing flow–volume; V′maxFRC, maximal flow at functional residual capacity; X5, reactance at 5 Hz; 2W, two-week follow-up; 4W, four-week follow-up. * Abnormal reduction defined as exceeding normal night-to-night variability in controls, as defined in the original study.

**Table 4:** EVI values reported in the included studies.

First Author Year	EVI in Healthy vs. Children with Asthma/Wheeze Median (IQR)	EVI in Treatment Response Median (IQR)	EVI in Acute Airway Obstruction Median (IQR)
Burman et al. 2022, [16]	NA	Baseline: 17.5 (15.2–18.9) * 43 days after ICS initiation: 18.4 (17.2–30.0) * 68 days after ICS initiation: 17.9 (17.1–19.6) *	NA
Seppä et al. 2020, [11]	2 days to discharge: 12.4 (7.7) 1 day to discharge: 14.9 (5.6) Controls: 17.4 (3.8)	NA	1 day to discharge: 14.9 (5.6) 2W: 16.4 (4.4) 4W: 17.0 (5.5) EVI in children with no auscultation findings: 17.5 (4.9) * EVI in children with wheeze and/or rales and crackles: 11.4 (6.8) * EVI in children with prolonged expiration only: 15.6 (7.4) *
Seppä et al. 2020, [17]	Not numerically reported for children with wheeze; EVI presented graphically as boxplots only. Controls: 17.3 (5.7)	Not numerically reported; EVI presented graphically as boxplots only.	Not numerically reported: EVI presented graphically only.

**Abbreviations:** EVI, expiratory variability index; ICS, inhaled corticosteroids; IQR, interquartile range; NA, not available; 2W, two-week follow-up; 4W, four-week follow-up. In some studies, dispersion was reported as IQR width rather than lower-upper values. * Dispersion measures were not explicitly defined in the study.

## Data Availability

No new data were created. For inquiries of the review process, please contact Fanny Kullberg (fanny.kullberg@gmail.com).

## Supplementary Materials

The following supporting information can be downloaded at: https://www.mdpi.com/article/10.3390/children13020193/s1. References [20,21,22,23,24,25,26,27,28,29,30,31,32,33,34].

## Abbreviations

The following abbreviations are used in this manuscript:

AHR	airway hyperresponsiveness
AUC	area under the curve
BD	bronchodilator
BD+	children that used bronchodilator
BD–	children that did not use bronchodilator
CI	confidence interval
EVI	expiratory variability index
FeNO	fractional exhaled nitric oxide
ICS	inhaled corticosteroids
IOS	impulse oscillometry
IP	impedance pneumography
IQR	interquartile range
NOS	Newcastle-Ottawa Scale
PRISMA	Preferred Reporting Items for Systematic reviews and Meta-Analyses
RoB	risk of bias
R5	resistance at 5 Hz
TBFV	tidal breathing flow–volume
V’maxFRC	maximal flow at functional residual capacity
X5	reactance at 5 Hz
2 W	two-week follow-up
4 W	four-week follow-up
