# Supplementary material for: Impedance Pneumography for Diagnosing and Monitoring Asthma in Young Children: A Systematic Review
Source: Children (Basel). 2026 Jan 29;13(2):193. doi: 10.3390/children13020193 (PMC12939778; doi:10.3390/children13020193)
Supplement: Supplementary file 1 [file children-13-00193-s001.zip › children-4054147-supplementary.pdf]

SUPPLEMENTARY MATERIAL

FULL TEXT REVISION – EXCLUDED ARTICLES

Article	Reason for exclusion
Asai, H., Furuya, N., Ando, T., Asai, M., Yoshihara, S., & Ichimura, T. (1991). Breathing Patterns During Sleep in Stable Asthmatic Children. *Journal of Asthma*, *28*(4), 265–272.	Did not meet age criteria
Asai, H., Onisawa, S., Furuya, N., Abe, T., & Ichimura, T. (1990). Breathing Patterns in Asthmatic Children During Attack. *Journal of Asthma*, 27(4), 229–236.	Did not meet age criteria
Bloise S., Nenna R., Petrarca L., et al THE ROLE OF RESPIRATORY FUNCTION TESTS IN INFANTS WITH SUSPECTED AIRWAY OBSTRUCTION. *Eur. Respir. J.* 2023;62(Supplement 67):PA3984.	Conference Abstract
Conti M.G., Petrarca L., Mancino E., et al The use of the tidal breathing flow-volume loop (TBFVL) in full-term newborns as a predictive tool for the risk of lower respiratory tract pathologies: an observational study. *Eur. Respir. J.* 2024;64(Supplement 68):PA4950.	Conference Abstract
Dahl, M. and Välimäki, I. (1967), Impedance Pneumography in Newborn Infants. *Acta Pædiatrica*, 56: 105-105.	No focus on asthma or wheeze
De Gouveia Beinelo P., Jesson K., Silva Sena C., et al Lung function at 6 weeks of age is associated with the risk of developing bronchiolitis in infants born to mothers with asthma during pregnancy. *Respirology* 2018;23(Supplement 1):25.	Conference Abstract
Di Mattia G., Petrarca L., Mancino E., et al Association between airway dimension, the clinical severity of bronchiolitis and the development of pre-school wheezing. *Eur. Respir. J.* 2023;62(Supplement 67):PA2085.	Conference Abstract
Ghalibafsabbaghi B., Raj D., Lodha R., Kabra S.K. Assessment of bronchodilator response in preschool children by pulmonary function tests. *Indian Pediatr.* 2013;50(10):957-960.	No use of impedance pneumography
Gracia-Tabuenca J, Seppä VP, Jauhiainen M, Kotaniemi-Syrjänen A, Malmström K, Pelkonen A, et al. Tidal breathing flow volume profiles during sleep in wheezing infants measured by impedance pneumography. *J Appl Physiol.* 2019 May 1;126(5):1409–18.	Did not report relevant outcome
Gracia-Tabuenca J, Seppä VP, Jauhiainen M, Paassilta M, Viik J, Karjalainen J. Tidal breathing flow profiles during sleep in wheezing children measured by impedance pneu-mography. *Respir Physiol Neurobiol.* 2020 Jan;271:103312.	Did not report relevant outcome
Kim Y.H., Park M., Kim S.Y., et al Tidal Breathing Analysis as a Prognostic Index for Airway Obstruction Trajectory and Asthma in Preterm Infants. *Lung* 2024;202(6):801-807.	No use of impedance pneumography
Malmberg LP, Seppä VP, Kotaniemi-Syrjänen A, Malmström K, Kajosaari M, Pelkonen AS, et al. Measurement of tidal breathing flows in infants using impedance pneumography. *Eur Respir J.* 2017 Feb;49(2):1600926.	Did not meet methodological criteria
Scaparrotta A., Verini M., Consilvio N.P., et al Allergological and lung function evaluation in wheezing infants: What usefulness?. *Paediatr. Respir. Rev.* 2010;11(SUPPL. 1):S79.	Conference Abstract
Seppä V.; Milagro J.; Pelkonen A.S.; Gil E.; Lazaro J., et al. Nocturnal variabilities of tidal airflow and heart rate show mutual association in young children with asthma symptoms. Oral Abstract Session OAS. *Allergy.* 2017;72(S103):3–127.	Conference Abstract
Seppa V.-P., Paassilta M., Gracia J., et al Tidal breathing variability during sleep is a sensitive marker of disease control in small children with recurrent wheeze. *Eur. Respir. J.* 2018;52(Supplement 62):no pagination.	Conference Abstract

Seppä VP; Pelkonen AS; Kotaniemi-Syrjänen A; Mäkelä MJ; Viik J; Malmberg LP. Tidal breathing flow measurement in awake young children by using impedance pneumography. *J Appl Physiol* (1985) Dec 2013;115(11):1725-31.	Wrong intervention
Seppa V.-P., Pelkonen A.S., Kotaniemi-Syrjanen A., Viik J., Makela M.J., Malmberg L.P. Erratum: Tidal flow variability measured by impedance pneumography relates to childhood asthma risk (European Respiratory Journal (2016) 47 (1687-1696)). *Eur. Respir. J.* 2016;48(1):285.	Correction/Erratum to published article
Seppä VP; Pelkonen AS; Kotaniemi-Syrjänen A; Viik J; Mäkelä MJ; Malmberg LP. Tidal flow variability measured by impedance pneumography relates to childhood asthma risk. *Eur Respir J.* Jun 2016;47(6):1687-96.	Wrong comparator
Seppa V.-P., Turkalj M., Hult A., Vuljanko I.M., Plavec D. Expiratory variability index (EVI) in young children during asthma exacerbation. *Eur. Respir. J.* 2019;54(Supplement 63):no pagination.	Conference Abstract
